# Physical Activity Level of Urban Pregnant Women in Tianjin, China: A Cross-Sectional Study

**DOI:** 10.1371/journal.pone.0109624

**Published:** 2014-10-06

**Authors:** Yan Zhang, Shengwen Dong, Jianhua Zuo, Xiangqin Hu, Hua Zhang, Yue Zhao

**Affiliations:** 1 School of Nursing, Tianjin Medical University, Tianjin, China; 2 Obstetrics and Gynecology Department, Wangdingdi Hospital, Tianjin, China; 3 Tianjin Central Hospital of Gynecology and Obstetrics, Tianjin, China; University of Tolima, Colombia

## Abstract

**Objective:**

To determine the physical activity level and factors influencing physical activity among pregnant urban Chinese women.

**Methods:**

This prospective cross-sectional study enrolled 1056 pregnant women (18–44 years of age) in Tianjin, China. Their socio-demographic characteristics were recorded, and the Pregnancy Physical Activity Questionnaire was used to assess their physical activity during pregnancy. The data were analyzed by multinomial logistic regression with adjustment for potential confounders.

**Results:**

Median total energy expenditure of pregnant women in each of the three trimesters ranged from 18.50 to 21.90 metabolic equivalents of task (METs) h/day. They expended 1.76–1.85 MET h/day on moderate and vigorous activities and 0.11 MET h/day on exercise. Only 117 of the women (11.1%) met the international guideline for physical activity in pregnancy (≥150 min moderate intensity exercise per week). The most frequent reason given for not being more physically active was the fear of miscarriage. Higher education level (OR: 4.11, 95% CI: 1.59–10.62), habitual exercise before pregnancy (OR: 2.14, 95% CI: 1.39–3.28), and husbands who exercised regularly (OR: 2.21, 95% CI: 1.33–3.67) significantly increased the odds of meeting the guideline (p<0.001). A low pre gravid body mass index (OR: 0.42, 95% CI: 0.20–0.87) significantly decreased the odds (*p*<0.001).

**Conclusions:**

Few urban Chinese pregnant women met the recommended physical activity guideline. They also expended little energy exercising. Future interventions should be based on the clinic environment and targeting family members as well as the subjects. All pregnant women should be targeted, not just those in high-risk groups.

## Introduction

Pregnancy is an important period for both women and their children, and excessive gestational weight gain (GWG) is a common phenomenon globally because many women decrease their physical activity during pregnancy [1]. Excessive GWG can lead to pregnancy complications such as gestational diabetes, and may also promote negative health outcomes for the child such as excess weight or obesity [2]. Physical activity during pregnancy may reduce the risk of prenatal complications and prevent excessive GWG [3], [4]; however, the incidence of low levels of physical activity during pregnancy is increasing globally because many women stop exercising and choose to rest and relax instead [5].

In 2002, the American College of Obstetricians and Gynecologists (ACOG) updated their guidelines on exercise for pregnant women and recommended moderate intensity exercise for at least 30 min per day on most days of the week [6]. However, the findings of studies on the levels of physical activity in pregnant women in Western countries are inconsistent. In 2012, a survey of 3482 pregnant women in Norway showed that only 14.6% followed the recommended guideline (≥3 times a week, 20 min per session, moderate intensity) at 17–21 weeks gestation [7], but in 1991 and 1992, a British study of 9889 pregnant women reported that approximately 50% participated in vigorous activity for at least 3 h per week at 18 and 32 weeks of gestation [8]. In 2004, a report from the UK showed that 39% of pregnant women who exercised regularly before pregnancy stopped exercising after becoming pregnant [9]. Another study showed that 21.5% healthy pregnant women in Ireland met the recommended guideline [10]. A cohort study of 1280 pregnant American women 1999–2006 revealed that only 22.9% met the ACOG recommended guidelines [11]; however, another study, conducted in the United States in 2013, found that 94.5% of women (52/55) at 18 weeks of pregnancy accumulated ≥150 min of moderate-to-vigorous activity weekly [12]. Various factors have been shown to influence the amount of physical activity or exercise taken by pregnant women, including educational level [13], pre gravid body mass index (BMI) [4], discomfort symptoms [4], [7], age [14], unemployment [14], and their husband's exercise habits [14]. Studies conducted in Brazil and Australia found that the main reasons given by pregnant women for not being more physically active were incontinence, discomfort, lack of time, and feeling tired [15], [16].

Most previous studies included mainly Caucasian women [7], [8], [9], [10], [11], [12] and only a few were carried out in Asian populations including Chinese populations [17]. China is the most populous country in the world. At the present time, excessive GWG is a public health problem in China [3]. A cohort study of 862 pregnant Chinese women found that more than 50% of participants experienced excessive weight gain during pregnancy [3]. Most pregnant women decreased their physical activity, reduced house-work or even quit their jobs as soon as they became pregnant. At the same time they increased their sedentary activities [17]. The prevalence of adherence to appropriate levels of physical activity, as defined by the international recommended guideline, by pregnant women in China remains unknown. Moreover, the factors that influence physical activity in this population have not yet been explored. Tianjin, the third largest city in China, is located in the eastern coastal area in China. In recent years, Tianjin has experienced rapid economic development and urbanization in line with overall national development. These transitions maybe influence physical activity behavior and related perceptions [18]. This population-based cross-sectional study conducted in Tianjin, evaluated physical activity level during pregnancy and factors that influence physical activity during pregnancy. The objective was to identify barriers that prevent pregnant Chinese women from exercising.

## Material and Methods

### Ethics

The study was approved by the Ethics Committee of the Tianjin Medical University (TMUhMEC 2012010). All participants were informed of the purposes and procedures of the study and all provided both verbal and written consent.

### Study design

For this population-based cross-sectional study, 1100 pregnant women in any of the three trimesters were recruited consecutively between February 2012 and February 2013 at two community hospitals, one obstetrics and gynecology hospital, and one general hospital during prenatal care visits. All study centers were located in urban districts of Tianjin, China. Pregnant women who had medical or obstetric complications that limited their physical activity or who were unwilling to take part in the study were excluded. A total of 40 subjects were excluded due to missing exercise-related information, and another four subjects who suffered discomfort while filling out the questionnaires were also excluded; thus 1056 pregnant women were included in the current analysis.

At the time of recruitment, just after their prenatal check, the women were informed about the purposes and procedures of this study. They were then informed about the questionnaires that would be administered by the investigators if they consented to participate. All subjects were assured that the questionnaires would be anonymous, therefore ensuring their privacy. After this introduction, the women were invited to participate in the study and after giving oral and written consent, the participants completed two questionnaires over a period of 15 min in a quiet place in the clinic. The questionnaires were then placed in a sealed box that could only be opened by the researchers.

### Questionnaires and measurements

The socio-demographic information was divided into two sections. The first included questions about the subject's socio-demographic characteristics (e.g., age, education level, pre gravid and current work status). The second section collected general information on exercise characteristics including whether the subject had ever received exercise advice from a health professional during pregnancy, what the subject's prepregnancy exercise habits were, and what the husband's exercise habits were. A baseline BMI was calculated from pre gravid weight and height, which were recorded in the medical records.

In 2004, Chasan-Taber *et al*. [19] developed the Pregnancy Physical Activity Questionnaire (PPAQ) to assess physical activity during pregnancy. This questionnaire includes 32 activities: household/caregiving activities (thirteen activities), occupational activities (five activities), sports/exercise activities (eight activities), transportation activities (three activities), and inactivity (three activities). The PPAQ measures the frequency and duration of activities, and gives an intensity value to each activity. The activities can be analyzed by type, by intensity or for the total energy expenditure. The PPAQ was validated by 7 days of testing with accelerometer measurements in a group of 54 pregnant women. The PPAQ was found to be a reliable measure of the physical activity of pregnant women in Australia [19], France [20], Japan [21] and Vietnam [22].

Few structured and validated questionnaires that can measure the physical activity of pregnant women have been developed in China [23]. Most available questionnaires do not include household or childcare activities, which make up a large part of physical activity during pregnancy [19]. The existing questionnaires cannot calculate the energy consumption of physical activity in pregnant women [23].

Regarding the accuracy and validity of PPAQ, with the approval of Chasan-Taber *et al*., we translated the PPAQ into Chinese, adapted it to culture conditions in China, and then translated it back into English. Five experts (including two chief physicians and one chief nurse majoring in obstetrics, one professor specializing in physical activity and sports, and one nursing educational specialist) were then invited to analyze the face and content validity of the Chinese version of the PPAQ. The resulting questionnaire lacked three items that were present in the Western version. These were, “holding children”, “mowing the lawn by riding a mower”, and “mowing the lawn by using a walking mower, raking, and gardening”. Two items were added, namely, “riding a bicycle” and “going up and down the stairs”. The modified Chinese version of the PPAQ thus included 31 items for measuring physical activity during pregnancy. They comprise household/caregiving activities (ten activities), occupational activities (five activities), sports/exercise activities (eight activities), transportation activities (five activities), and inactivity (three activities).

Following its development, 30 pregnant Chinese women were surveyed using the revised questionnaire. They were then asked to wear Live Pod-02 human motion energy consumption meters to monitor their energy consumption for 7 days. At the end of the 7 days, the correlation between the questionnaire results and the meter results was analyzed. The content validity and test-retest reliability of the questionnaire were 0.940 and 0.944, respectively, while the correlation coefficient between meter and questionnaire results was 0.768 [23].

The energy expenditure for each activity was then calculated by multiplying the self-reported time spent in each activity per day by the activity intensity. The total activity expenditure was expressed as metabolic equivalents of task (METs) h per day, obtained by summing all activities. Each activity was classified according to intensity (sedentary, <1.5 METs; light, ∼1.5 METs; moderate, ∼3.0 METs]; and vigorous, ∼6.0 METs) and type (i.e., household/caregiving, occupational, and sports/exercise). The average number of METs h per day for each intensity level and activity type was calculated [19]. The total time spent in sports/exercise activities per week was calculated by multiplying the self-reported time spent on each sports/exercise activity per day by the activity intensity based the on PPAQ. Pregnant women who reported more than 2 h moderate intensity sports/exercise activity per week were deemed to meet the international guideline [6], [7], [12].

### Statistical analysis

The subjects who met the guideline, socio-demographic characteristics, exercise-related information, and energy expenditure were expressed as numbers, percentages, and means. The significance of differences in the results for continuous and categorical variables among the women within each trimester group were determined by one-way ANOVA and the chi-square test, respectively. Multinomial logistic regression analysis was used to estimate the odds ratios (ORs) and 95% confidence intervals (CIs) of meeting the activity guideline. The results were presented as crude and adjusted ORs with 95% CIs. *p*-values of <0.05 were considered to indicate statistical significance. All data were analyzed using SPSS 17.0 (SPSS, Chicago, IL).

## Results

Of these participants, 172 (16.3%), 512 (48.5%), and 372 (35.2%) were in the first, second, and third trimester, respectively. The mean age of the participants was 27.89±3.53 years. The other socio-demographic characteristics of the subjects are presented in Table1.

**Table 1:** Socio-demographic of pregnant women n (%).

Characteristic		Overall	1^st^ trimester	2^nd^ trimester	3^nd^ trimester
**N**		1056 (100.0)	172 (16.3)	512 (48.5)	372 (35.2)
**Age**		27.89±3.53	28.28±2.65	28.28±3.14	27.18±4.23
**Race**	Han Chinese	1002 (95.0)	160 (93.0)	482 (94.1)	360 (96.8)
	Others	54 (5.0)	12 (7.0)	30 (5.9)	12 (3.2)
**Currently employed**		590 (55.9)	132 (76.7)	318 (62.1)	140 (37.6)
**Education**	Lower than high school	180 (17.0)	8 (4.7)	89 (17.4)	83 (22.3)
	High school graduate	424 (40.2)	64 (37.2)	206 (40.2)	154 (41.4)
	College or higher degree	452 (42.8)	100 (58.1)	217 (42.4)	135 (36.3)
**Monthly household income** (RMB)	≤3500	368 (34.9)	28 (16.3)	144 (28.2)	196 (52.7)
	∼3501	274 (25.9)	64 (37.2)	136 (26.6)	74 (19.9)
	∼5001	338 (32.0)	70 (40.7)	192 (37.5)	76 (20.4)
	≥10,000	76 (7.2)	10 (5.8)	40 (7.9)	26 (7.0)
**Pre-gravid health status**	Very healthy	422 (40.0)	68 (39.5)	212 (41.4)	144 (38.7)
	Healthy	500 (47.3)	90 (52.3)	244 (47.7)	166 (44.6)
	General	134 (12.7)	14 (8.1)	56 (10.9)	62 (16.7)
**Pre-gravid work status (employed)**		750 (71.0)	136 (79.1)	361 (70.5)	253 (68.0)
**Pre-gravid BMI (kg/m^2^)**	Low (≤18.4)	164 (15.5)	24 (14.0)	72 (14.1)	68 (18.3)
	Normal (∼18.5)	764 (72.4)	122 (70.9)	386 (75.4)	356 (95.7)
	Overweight (∼25.0)	128 (12.1)	26 (15.1)	54 (10.5)	48 (12.9)
**Previous live births**	0	946 (89.6)	168 (97.7)	470 (91.8)	306 (82.2)
	1	100 (9.5)	2 (1.2)	36 (7.0)	62 (16.7)
	2	10 (0.9)	2 (1.2)	4 (0.8)	4 (1.1)
**Discomfort symptoms (yes)**		792 (75.0)	132 (76.7)	348 (68.0)	312 (83.9)

Of the 1056 participants, 87 (8.2%) had received advice about physical activity from a medical professional, and 786 (74.4%) women reported that they had become progressively less active during their pregnancy. In general, the participants considered their husband to be the most important person influencing their decision about exercise. Walking slowly was the most popular exercise. The most common reason given for not engaging in more physical activity during pregnancy was the fear of miscarriage (Table 2).

**Table 2:** Exercise-related characteristics of pregnant women (N = 1056) n (%).

Characteristic		Overall	1^st^ trimester	2^nd^ trimester	3^nd^ trimester
**Exercise advices from medical professional**		87 (8.2)	17(9.9)	34 (6.6)	36(9.7)
**Habitually exercise before pregnancy**		376 (35.6)	70 (40.7)	174 (34.0)	132 (35.5)
**Self-evaluation of physical activity level**	Low	448 (42.4)	66 (38.4)	217 (42.4)	165 (44.4)
	Moderate	600 (56.8)	103 (59.9)	291 (56.8)	206 (55.4)
	High	8 (0.8)	3 (1.7)	4 (0.8)	1 (0.3)
**Self-evaluation of physical activity changes after pregnancy**	Less active	786 (74.4)	129 (75.0)	381 (74.4)	276 (74.2)
	Increased activity	28 (2.7)	2 (1.2)	14 (2.7)	12 (3.2)
	No change	242 (22.9)	41 (23.8)	117 (22.9)	84 (22.6)
**Having to go with husband who habitually exercises**		820 (77.7)	139 (80.8)	398 (77.7)	283 (76.1)
		698 (66.1)	111 (64.5)	343 (67.0)	244 (65.6)
**Sources of information about exercise**	Books and newspapers	662 (62.7)	120 (69.7)	298 (58.3)	244 (65.6)
	Friends or relatives	346 (32.8)	68 (39.5)	178 (34.8)	100 (26.9)
	Multi-media	512 (48.4)	100 (58.2)	262 (51.2)	150 (40.3)
	Medical institutions	102 (9.7)	0 (0.0)	56 (10.9)	46 (12.4)
	Public announcement	142 (13.4)	52 (30.2)	58 (11.3)	32 (8.6)
**Desirable sources of information about exercise**	Medical institutions	554 (52.5)	90 (52.3)	278 (54.3)	186 (50.0)
	Public announcement	274 (25.9)	68 (39.6)	100 (19.5)	106 (28.5)
	Multi-media	192 (18.1)	22 (12.8)	102 (20.0)	68 (18.3)
	Books and newspapers	118 (11.2)	16 (9.3)	60 (11.7)	42 (11.3)
	Friends or relatives	126 (11.9)	12 (7.0)	74 (14.5)	40 (10.7)
**The person deciding about the exercise**	Husband or partner	848 (80.3)	156 (90.7)	436 (85.2)	256 (68.8)
	Medical staff	334 (31.6)	50 (29.1)	240 (46.9)	44 (11.8)
	Other family members or friends	300 (28.6)	60 (34.9)	116 (22.7)	124 (33.3)
**The preferred exercise**	Walking slowly	1032 (98.0)	172 (100.0)	506 (98.8)	354 (95.2)
	Prenatal exercise class	324 (30.7)	60 (34.9)	180 (35.2)	84 (22.6)
	Swimming	30 (2.8)	6 (3.5)	16 (3.1)	8 (2.2)
	Climb hill	18 (1.7)	4 (2.3)	8 (1.6)	6 (1.6)
	Dancing	10 (0.9)	2 (1.2)	2 (0.4)	6 (1.6)
	Walking quickly	8 (0.8)	0 (0.0)	4 (0.8)	4 (1.1)
	Running slowly	6 (0.5)	2 (1.2)	2 (0.4)	2 (0.5)
	Running quickly	3 (0.3)	0 (0.0)	3 (0.6)	0 (0.0)
**Reasons hindering exercise activities**	Fear of miscarriage	704 (66.7)	138 (80.2)	390 (76.2)	176 (47.3)
	Discomfort	370 (35.0)	68 (39.5)	216 (42.2)	86 (23.1)
	Lacking exercise knowledge	264 (25.0)	38 (22.1)	170 (33.2)	56 (15.1)
	Too tired	244 (23.1)	48 (27.9)	82 (16.0)	114 (30.6)
	Having no exercise habits	150 (14.2)	14 (8.1)	82 (16.0)	54 (14.5)
	Lack of venues or equipment	120 (11.4)	10 (5.8)	92 (18.0)	18 (4.8)
	Having no time	102 (9.7)	24 (14.0)	50 (9.8)	28 (7.5)
	Having no one to go with	90 (8.5)	4 (2.3)	52 (10.2)	34 (9.1)
	Dislikes exercise	56 (5.3)	10 (5.8)	24 (4.7)	22 (5.9)
	Weight gain	54 (5.1)	2 (11.6)	30 (5.9)	22 (5.9)

Based on the PPAQ, 117 (11.1%) women met the guideline. The rates of meeting the guideline did not differ significantly among the three trimesters (Table 3). Women in the first trimester had significantly higher median total energy expenditure (21.90 MET h/day) than women in the second and third trimesters (19.44 and 18.50 MET h/day, respectively; *p*<0.001). The median, moderate, and vigorous intensity expenditures during the three trimesters ranged from 1.75 to 1.86 MET h/day, and the median energy expenditure on sports/exercise for all subjects was 0.11 MET h/day. As shown in Table 4, sedentary activities accounted for more than half of the energy expenditure of all subjects (52.7–57.6%). By contrast, exercise accounted for only 1.7–2.4% of the energy expenditure for all subjects (Table 4).

**Table 3:** Physical activities during pregnancy.

Characteristic	1^st^ trimester (≤13 weeks)			2^nd^ trimester (14–27 weeks)			3^rd^ trimester (≥28 weeks)				
	Mean^a^	Median^a^	25^th^, 75^th^ percentile	Mean^a^	Media^a^	25^th^, 75^th^ percentile	Mean^a^	Median^a^	25^th^, 75^th^ percentile	*H*	*P value* b
Meet the guideline *n* (%)	20 (11.6)			58 (11.3)			39 (10.5)			0.219	0.896
Total energy expenditure per day	23.98	21.90	14.78, 28.04	20.41	19.44	12.31, 27.29	20.11	18.50	12.21, 26.71	22.000	0.000
By activity intensity											
Sedentary	13.82	11.74	7.70, 16.71	11.74	9.85	7.08, 16.4	10.59	9.58	6.63, 16.55	31.256	0.000
Light	5.76	5.29	2.61, 9.31	5.85	4.68	2.50, 7.93	6.03	4.53	2.53, 7.20	2.677	0.262
Moderate	4.28	1.86	0.86, 4.58	2.78	1.75	0.75, 4.19	3.45	1.75	0.75, 3.86	22.787	0.000
Vigorous	0.11	0.00	0.00, 0.00	0.02	0.00	0.00, 0.00	0.04	0.00	0.00, 0.00	10.611	0.005
By type of activity											
Household/caregiving	3.09	3.03	1.21, 5.33	3.75	3.03	1.25, 5.74	5.34	3.03	1.28, 5.74	41.139	0.000
Occupational	8.23	6.00	0.00, 10.15	5.49	2.06	0.00, 10.15	4.02	0.20	0.00, 10.15	73.347	0.000
Sports/exercise	0.57	0.11	0.00, 0.51	0.34	0.11	0.00, 0.36	0.43	0.11	0.00, 0.35	41.326	0.000
Transportation	3.89	2.75	0.38, 4.09	3.02	2.25	1.03, 4.00	2.35	2.00	1.12, 3.63	42.519	0.000

a MET-hours/day.

b Kruskal–Wallis test of differences in ranks.

**Table 4:** Contribution of each type of activity to the total expenditure across different trimesters.

Characteristics	1^st^ trimester	2^nd^ trimester	3^rd^ trimester
	(≤13 weeks) (%)	(14–27 weeks) (%)	(≥28 weeks) (%)
Total energy expenditure	100.0	100.0	100.0
Sedentary	57.7	57.6	52.7
Light	24.0	28.7	30.0
Moderate	17.8	13.6	17.1
Vigorous	0.5	0.1	0.2
Total energy expenditure^a^			
Household/caregiving	12.9	18.4	26.6
Occupational	34.3	26.9	20.0
Sports/exercise	2.4	1.7	2.1
Transportation	16.2	14.8	11.7

a Sitting accounted for 34% to 40% of total energy expenditure in the three trimesters.

As shown in Table 5, after adjusting for a number of factors, several variables strongly influenced the ORs of meeting the guideline. Pregnant women who had college degrees or higher education were more likely to meet the guideline (OR: 4.11, 95% CI: 1.59–10.62). However, having a low pre gravid BMI decreased the ORs of meeting the guideline (OR: 0.42, 95% CI: 0.20–0.87). Women who exercised regularly before pregnancy had an increased likelihood of meeting the guideline (OR: 2.14, 95% CI: 1.39–3.28). The odds of meeting the guideline were higher in women with a self-evaluation of current physical activity as moderate (OR: 2.43, 95% CI: 1.53–3.86) or high (OR: 9.49, 95% CI: 1.77–50.88), and those having a husband who exercised habitually (OR: 2.21, 95% CI: 1.33–3.67). Receiving advice from medical professionals did not increase the odds (OR: 0.74, 95% CI: 0.19–2.83) (Table 5).

**Table 5:** Association between participants' characteristics and the odds of meeting the guideline.

*Characteristics*		*n (%)*	*OR* (*95% CI*)	
			Unadjusted	Adjusted
**Age**		27.89±3.53	1.04 (0.99, 1.10)	1.02 (0.95, 1.10)
**Race**	Minority	54 (5.0)	0.30 (0.07, 1.23)	0.26 (0.06, 1.16)
**Currently employed**	Working	590 (55.9)	1.82 (1.21, 2.74)	1.10 (0.65, 1.87)
**Education**	Lower than high school	180 (17.0)	1.0 (Ref)	1.0 (Ref)
	High school graduate	424 (40.2)	1.93 (0.92, 4.06)	2.24 (0.95, 5.25)
	College or higher degree	452 (42.8)	3.42 (1.67, 7.02)	4.11 (1.59, 10.62)
**Monthly household income**	≤3500	368 (34.9)	0.41 (0.20, 0.85)	0.71 (0.31, 1.63)
(RMB)	∼3501	274 (25.9)	0.91 (0.45, 1.84)	1.30 (0.60, 2.83)
	∼5001	338 (32.0)	0.70 (0.35, 1.40)	0.70 (0.33, 1.51)
	≥10000	76 (7.2)	1.0 (Ref)	1.0 (Ref)
**Previous live births**	0	946 (89.6)	0.52 (0.11, 2.46)	0.64 (0.11, 3.68)
	1	100 (9.5)	0.30 (0.05, 1.70)	0.51 (0.08, 3.39)
	2	10 (0.9)	1.0 (Ref)	1.0 (Ref)
**Pre-pregnancy work status**	Working	750 (71.0)	1.41 (0.90, 2.22)	0.76 (0.42,1.36)
**Pre-pregnancy health status**	Very healthy	422 (40.0)	1.0 (Ref)	1.0 (Ref)
	Healthy	500 (47.3)	0.83 (0.55, 1.25)	1.04 (0.66, 1.63)
	General	134 (12.7)	0.92 (0.50, 1.69)	1.03 (0.52, 2.04)
**Pre-gravid BMI (k** ***g/m^2^*** **)**	Low (≤18.4)	164 (15.5)	0.39 (0.19, 0.78)	0.42 (0.20, 0.87)
	Normal (∼18.5)	764 (72.4)	1.0 (Ref)	1.0 (Ref)
	Overweight (∼25.0)	128 (12.1)	0.44 (0.21, 0.93)	0.64 (0.16, 2.58)
**Discomfort symptoms**	Yes	792 (75.0)	0.83 (0.54, 1.28)	0.75 (0.49, 1.85)
**Stage of pregnancy**	First trimester	172 (16.3)	1.12 (0.63, 1.99)	0.95 (0.55, 2.09)
	Second trimester	512 (48.5)	1.09 (0.71, 1.68)	0.86 (0.52, 1.41)
	Third trimester	372 (35.2)	1.0 (Ref)	1.0 (Ref)
**Exercise before pregnancy**	Yes	376 (35.6)	2.33 (1.58, 3.44)	2.14 (1.39, 3.28)
**Self-evaluation of current physical activity level**	Low	448 (42.4)	1.0 (Ref)	1.0 (Ref)
	Moderate	600 (56.8)	1.94 (1.26, 2.96)	2.43 (1.53, 3.86)
	High	8 (0.8)	12.58 (3.00,52.58)	9.49 (1.77,50.88)
**Having to go with husband who habitually exercises**	Yes	820 (77.7)	1.20 (0.74, 1.94)	1.14 (0.69, 1.90)
	Yes	698 (66.1)	1.72 (1.10, 2.69)	2.21 (1.33, 3.67)
**Exercise advice from medical professional**	Yes	87(8.2)	0.51(0.24, 1.07)	0.74(0.19, 2.83)

## Discussion

Only 11.1% of the pregnant women who participated in this study met the international recommended guideline for physical activity during pregnancy. This rate is lower than the rates recorded in Ireland (21.5%) [10] and the United States (22.9% and 94.5%) [11], [12]. In addition, the subjects in this study expended less total energy during pregnancy (approximately 20 MET h/day) than pregnant women in the United States (25.4 MET h/day) [12], Australia (30.0 MET h/day) [13], and France (29.0 MET h/day) [20]. However, the contributions of specific activities to the total energy expenditure found in this study population were similar to the Australian and French study populations [13], [20].

The women in this study expressed concern about the safety of exercise, reporting that the primary reason for not exercising during pregnancy was fear of miscarriage. By contrast, women who participated in studies conducted in Brazil and Australia stated that their primary reasons for lack of exercise in pregnancy were feeling tired, lack of time, and incontinence [15], [16]. This difference may be related to traditional Chinese culture, which defines pregnancy as a vulnerable period that requires rest and protection. Therefore, to avoid spontaneous miscarriage and reduce pressure from family and friends, pregnant Chinese women tend to obey traditional taboos such as “no jumping”, “no moving heavy objects”, “no fast walking”, and “not too much walking” [24]. This may partly explain why the pregnant Chinese women reported that their most common form of exercise was slow walking, which is very different from pregnant British women, who report that fast walking, swimming, and prenatal exercise classes are their most popular forms of exercise [14]. The cultural taboos against exercise in pregnancy in China are exacerbated by the Family Planning Policy, which has greatly increased the focus of family members on the fetus and the pregnant woman [25]. As a result, it is believed that cutting back on exercise and even household/caregiving activities aids the growth of the fetus [24]. These cultural factors may explain why 74.4% of these study subjects become less physically active during pregnancy and why exercise contributed little to total energy expenditure. The fact that a large number of women in China stop exercising after becoming pregnant had been observed previously; moreover, more than half of Chinese women become overweight during pregnancy [3]. Evenson *et al*. have previously noted that the influence of different cultural beliefs on physical activity during pregnancy needs further exploration [26].

The present study showed that only 8.2% of the pregnant Chinese participants were given advice by medical professionals about physical activity. By contrast, an American study showed that 63% of pregnant women in the United States were given advice about physical activity during pregnancy and that more than half believed that such advice from doctors could improve their exercise behavior in pregnancy [27]. A recent systematic review of nine randomized controlled trials of behavioral interventions for improving physical activity among pregnant women suggests that medical providers play a key role in such interventions [28]. However, in this study, receiving advice from health professionals did not increase the rate at which the participants met the international guideline. A study in Taiwan found that individual counseling on diet and physical activity provided during routine clinic visits made the pregnant women healthier and more active and thus assisted with weight management during pregnancy [29]. Therefore, because nonsystematic medical advice may not be adequate for changing the exercise behaviors of pregnant women in China, a systematic clinic-based intervention protocol may be warranted.

Consistent with previous studies, the pregnant women who had higher educational levels were more active during their pregnancy than women with lower educational levels [4], [13]. This correlation may be related to the possibility that women with higher educational levels have more access to knowledge about physical activity during pregnancy and thus are more likely to exercise [13]. Moreover, the present study showed that women who exercised regularly and evaluated their current level of physical activity as moderate or high were more likely to meet the guideline. This finding suggests that this self-evaluation is relatively objective, and that habitual exercise before pregnancy can influence women to continue exercising during pregnancy. The latter observation has also been made previously [4].

A previous study in Australia showed that women with high pre gravid BMIs were less active during pregnancy than women with lower pre gravid BMIs [4]. By contrast, the present study showed that Chinese women with low pre gravid BMIs were less likely to meet the guideline than those with higher BMIs. Huang *et al*. [30] observed that a low weight before pregnancy also increased the risk of excessive GWG in women in Taiwan, which may reflect several cultural beliefs in China. First, traditional Chinese medicine holds that one is healthy when the *yin* and the *yang* are balanced, and that pregnancy can cause a harmful imbalance in the pregnant women's body [31]. To prevent this imbalance, Chinese women are encouraged to greatly increase their consumption of food and to decrease their physical activity [25]. This is exacerbated by the belief of the older women in Chinese families that it is necessary to gain weight during pregnancy. This belief reflects the experiences of these women with food shortages experienced in previous decades [29]. Notably, previous interventions for improving the physical activity of pregnant women mainly focused on high-risk groups such as obese women [28], but our observation that women with low pre gravid BMI were more likely not to meet the guideline than women with higher BMI indicates that future interventions should target all women, not just the high-risk women.

The present study also showed that pregnant women with husbands who exercised habitually were more likely to meet the guideline than women with less active husbands. The exercise levels of pregnant women correlated positively with their partner's physical activity levels. The observation that women living with an active partner may be more active during pregnancy than women without an active partner has been reported previously [14]. Thornton *et al*. [32] also posited that the husband is the most important influence on decisions about exercise in pregnancy because the husband provides the pregnant women with the most social support. In Chinese culture, antenatal taboos are mainly imparted by family members [25]. Further studies are required to determine whether maximizing the positive role of family members such as husbands will encourage Chinese women to increase their physical activity during pregnancy.

There were several limitations in present study. First, only one city was included, and all of the participants were urban residents. The study results may thus not be generalizable to all Chinese populations, including rural pregnant women. Studies examining the residents in multiple Chinese cities and in rural areas are warranted. Second, the reliability of self-reported measurements is lower than that of data obtained by accelerometry. However, the PPAQ is a validated tool for assessing the duration and intensity of exercise, and defining the types of physical activity that pregnant women engage in, and it is better than unstructured questions [19]. In addition, the questionnaire can collect data on some activities that could not be detected with accelerometry [33]. Third, data from this cross-sectional study do not illustrate exercise conditions throughout pregnancy. Therefore, longitudinal follow-up is warranted in future studies. Finally, although we explored a considerable number of factors influencing physical activity during pregnancy, we could not measure the influence of other factors such as social and cultural factors [26]. In the future, multicenter studies are needed for measuring additional factors influencing physical activity during pregnancy in China.

## Conclusions

Few urban Chinese pregnant women met the recommended physical activity guideline. They also expended little energy on exercise. Physical activity interventions that are conducted in the clinic environment and that target family members and social networks in addition to the pregnant woman herself, may improve the physical activity and health of pregnant Chinese women and should be explored. Moreover, pregnant women with low pre gravid BMIs should also be given as much attention in future interventions as women who are deemed to be at high risk.
